# Characterization of cardiac mechanics and incident atrial fibrillation in participants of the Cardiovascular Health Study

**DOI:** 10.1172/jci.insight.141656

**Published:** 2020-10-02

**Authors:** Ravi B. Patel, Joseph A. Delaney, Mo Hu, Harnish Patel, Jeanette Cheng, John Gottdiener, Jorge R. Kizer, Gregory M. Marcus, Mintu P. Turakhia, Rajat Deo, Susan R. Heckbert, Bruce M. Psaty, Sanjiv J. Shah

**Affiliations:** 1 Division of Cardiology, Department of Medicine, Northwestern University Feinberg School of Medicine, Chicago, Illinois, USA.; 2 College of Pharmacy, University of Manitoba, Winnipeg, Manitoba, Canada.; 3 Division of Cardiology, Department of Medicine, University of Maryland School of Medicine, Baltimore, Maryland, USA.; 4 Division of Cardiology, Department of Medicine, San Francisco Veterans Affairs Health Care System, San Francisco, California, USA.; 5 Division of Cardiology, Department of Medicine, UCSF, San Francisco, California, USA.; 6 Division of Cardiology, Veterans Affairs Palo Alto Health Care System, Palo Alto, California, USA.; 7 Division of Cardiology, University of Pennsylvania, Philadelphia, Pennsylvania, USA.; 8 Cardiovascular Health Research Unit, Departments of Medicine, Epidemiology, and Health Services, University of Washington, Seattle, Washington, USA.; 9 Kaiser Permanente Washington Health Research Institute, Seattle Washington, USA.

**Keywords:** Cardiology, Arrhythmias, Cardiovascular disease, Epidemiology

## Abstract

**BACKGROUND.** Left atrial (LA) and left ventricular (LV) remodeling are associated with atrial fibrillation (AF). The prospective associations of impairment in cardiac mechanical function, as assessed by speckle-tracking echocardiography, with incident AF are less clear.

**METHODS.** In the Cardiovascular Health Study, a community-based cohort of older adults, participants free of AF with echocardiograms of adequate quality for speckle tracking were included. We evaluated the associations of indices of cardiac mechanics (LA reservoir strain, LV longitudinal strain, and LV early diastolic strain rate) with incident AF.

**RESULTS.** Of 4341 participants with strain imaging, participants with lower LA reservoir strain were older, had more cardiometabolic risk factors, and had lower renal function at baseline. Over a median follow-up of 10 years, 497 (11.4%) participants developed AF. Compared with the highest quartile of LA reservoir strain, the lowest quartile of LA reservoir strain was associated with higher risk of AF after covariate adjustment, including LA volume and LV longitudinal strain (Hazard Ratio [HR], 1.80; 95% CI, 1.31–2.45; P < 0.001). The association of LA reservoir strain and AF was stronger in subgroups with higher blood pressure, NT-proBNP, and LA volumes. There were no associations of LV longitudinal strain and LV early diastolic strain rate with incident AF after adjustment for LA reservoir strain.

**CONCLUSION.** Lower LA reservoir strain was associated with incident AF, independent of LV mechanics, and with stronger associations in high-risk subgroups. These findings suggest that LA mechanical dysfunction precedes the development of AF. Therapies targeting LA mechanical dysfunction may prevent progression to AF.

**FUNDING.** This research was supported by contracts HHSN268201200036C, HHSN268200800007C, HHSN268201800001C, N01HC55222, N01HC85079, N01HC85080, N01HC85081, N01HC85082, N01HC85083, and N01HC85086 and grants KL2TR001424, R01HL107577, U01HL080295, and U01HL130114 from the NIH’s National Center for Advancing Translational Sciences, and National Heart, Lung, and Blood Institute (NHLBI), with additional contribution from the National Institute of Neurological Disorders and Stroke (NINDS). Additional support was provided by R01AG023629 from the National Institute on Aging (NIA). A full list of principal CHS investigators and institutions can be found at CHS-NHLBI.org.

## Abstract 2

Left atrial reservoir strain was independently associated with higher risk of atrial fibrillation in this community-based cohort of elderly adults.

## Graphical abstract

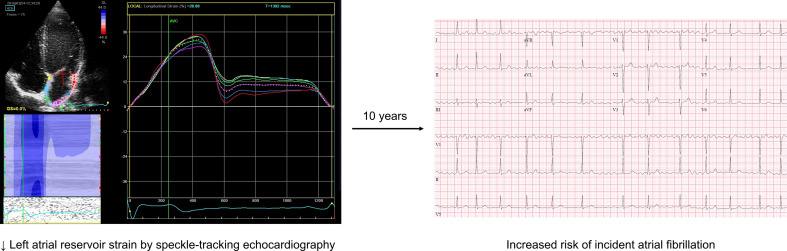

## Introduction

Atrial fibrillation (AF) burdens approximately 30 million individuals worldwide and, despite contemporary treatment strategies, is associated with substantial cardiovascular morbidity and mortality (1, 2). While multiple clinical risk factors are closely associated with incident AF, traditional clinical variables may incompletely define the myocardial substrate that drives AF to exist. The left atrial (LA) and left ventricular (LV) myocardium undergo a series of maladaptive changes before the development of clinical AF, including both structural and functional aspects of remodeling. Although several indices of structural remodeling of cardiac chambers are associated with incident AF (3–5), the myocardial substrate that leads to the maintenance of AF is not well understood (6).

Speckle-tracking strain echocardiography is an imaging technique that assesses myocardial tissue deformation and provides sensitive measures of myocardial function (7, 8). For example, LA reservoir strain assesses deformation of the LA during ventricular systole, serving as an aggregate measure of LA function during LA filling (9). In addition to functional assessment, strain imaging may be reflective of myocardial fibrosis (10), and upon ultrastructural analysis, strain abnormalities are associated with cardiomyocyte calcium-handling abnormalities (11). Therefore, echocardiographic strain measurement of cardiac chambers serves as a composite measure of myocardial structural and functional remodeling that may identify high-risk myocardial substrate for progression to AF. However, the temporal associations between these measures of cardiac mechanics as assessed by strain and development of AF have not been well described. We therefore evaluated the associations of multiple indices of LA and LV cardiac mechanics with incident AF in the Cardiovascular Health Study (CHS).

## Results

### Study participant characteristics.

**Figure 1:**
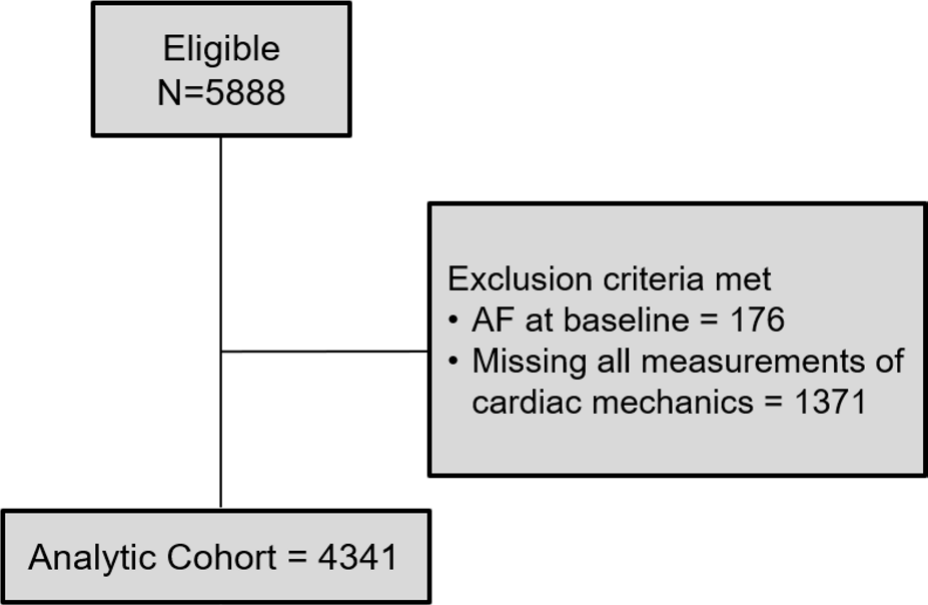
STROBE diagram of study inclusion.

Of the 5888 participants in CHS, 176 had AF at baseline and 1371 did not have adequate echocardiograms for speckle tracking of at least 1 cardiac chamber. The final analytic cohort consisted of 4341 participants (Figure 1). Characteristics of participants without AF at baseline who were excluded from the final analytic cohort compared with those included are displayed in Supplemental Table 1 (supplemental material available online with this article; https://doi.org/10.1172/jci.insight.141656DS1). The baseline clinical profile of participants by quartiles of LA reservoir strain, LV longitudinal strain, and LV early diastolic strain rate are shown in Table 1 and Supplemental Tables 2 and 3, respectively. Participants with lower absolute LA reservoir strain levels were older and more likely female. LA reservoir strain was significantly lower in Black participants compared with White participants at baseline (38.3% ± 16.1% versus 40.8% ± 15.3%, *P* = 0.006). Additionally, those with lower LA reservoir strain had higher baseline prevalence of hypertension, diabetes mellitus (DM), heart failure (HF), and prior myocardial infarction. Participants with lower LA reservoir strain had higher baseline BMI, low-density lipoprotein, triglycerides, glucose, N-terminal pro–B-type natriuretic peptide (NT-proBNP), soluble ST2 (sST2), galectin-3 (Gal-3), and high-sensitivity troponin T (hs-TnT) levels, and lower baseline estimated glomerular filtration rate (eGFR). The clinical and laboratory profiles of participants by LV longitudinal strain and LV early diastolic strain rate were generally consistent (Supplemental Tables 2 and 3).

**Figure 2:**
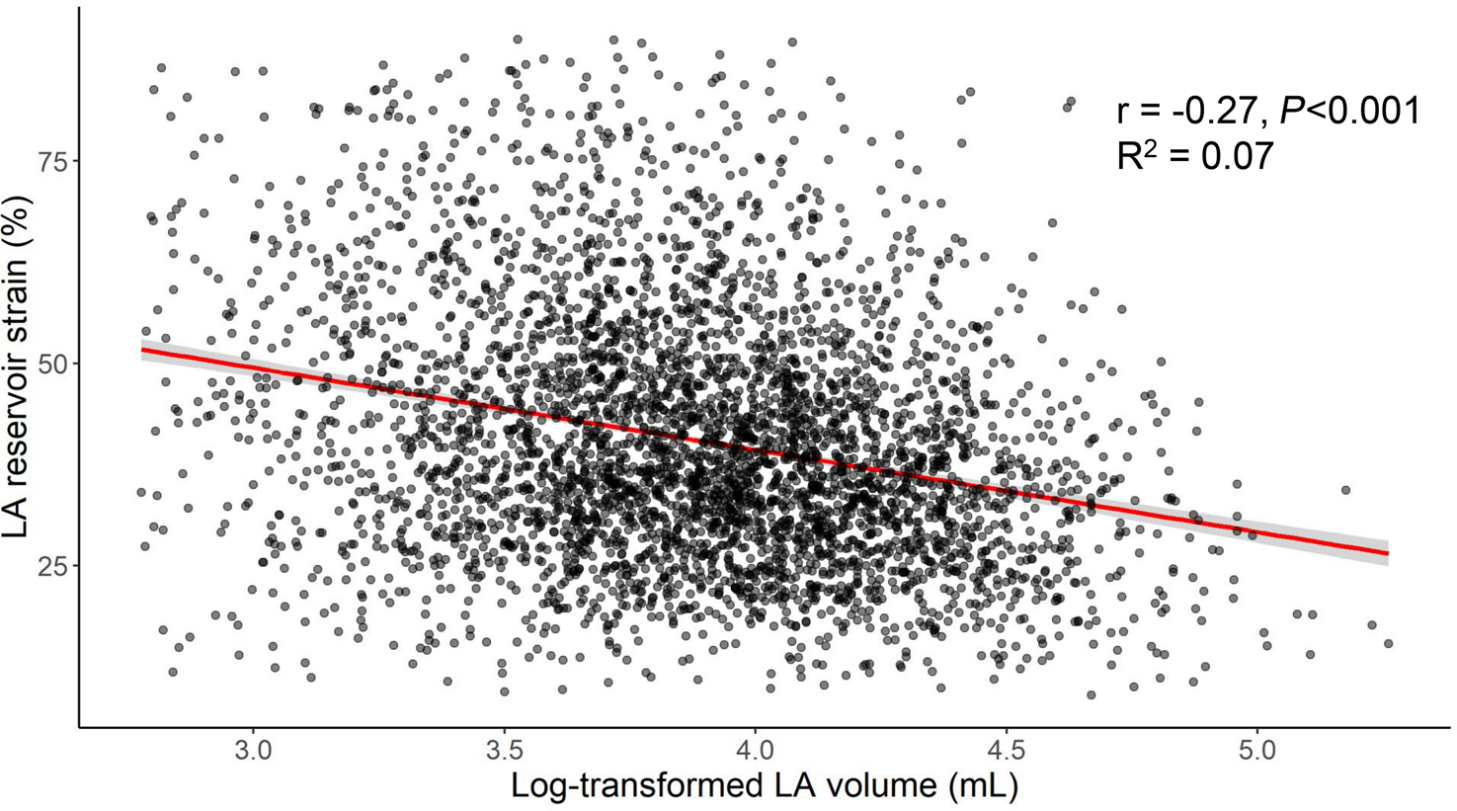
Association of LA volume and LA reservoir strain. Shown is a linear model (red) evaluating the association of log-transformed LA volume and LA reservoir strain. LA volumes were log transformed to maintain constant variance of residuals. LA, left atrial.

Participants with lower LA reservoir strain at baseline had lower LV longitudinal strain and LV early diastolic strain rate on raw analysis (Table 2). Additionally, participants with lower LA reservoir strain had higher LA volume (LAV), higher LV mass, and lower LV ejection fraction (LVEF) at baseline. There was a significant but modest correlation between higher LAV and lower LA reservoir strain (*r* = –0.27, *P* < 0.001) (Figure 2).

### Association of indices of cardiac mechanics and incident AF.

**Figure 3:**
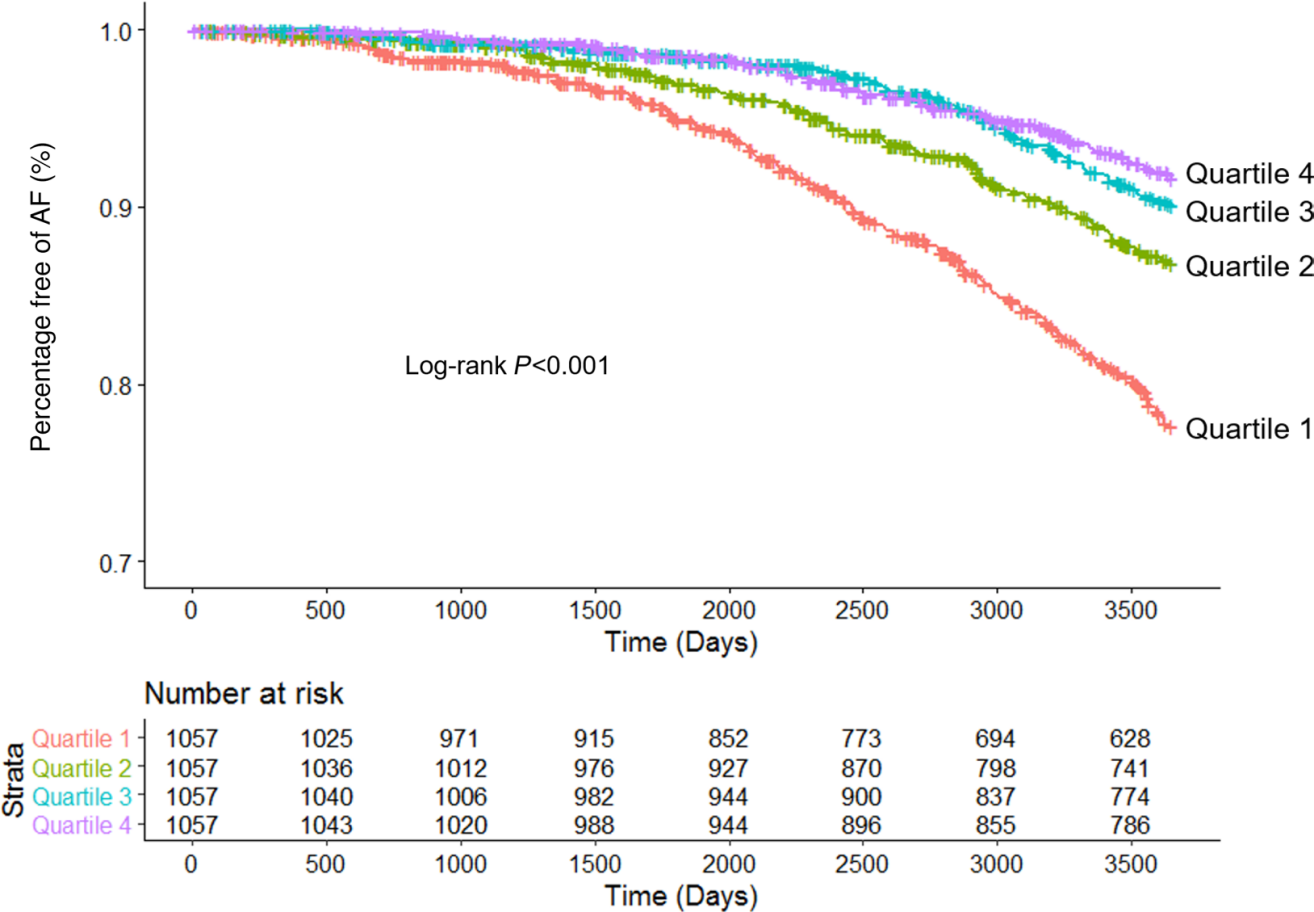
Risk of incident AF stratified by quartiles of LA reservoir strain. Shown are Kaplan-Meier curves depicting the percentage of participants free of AF over time by quartile of LA reservoir strain.

Over median follow-up of 10.0 years, 497 (11.4%) participants developed AF among the final analytic cohort (*n* = 4341). Compared with participants in the highest quartile of LA reservoir strain (Quartile 4), the lowest quartile of LA reservoir strain (Quartile 1) was independently associated with incident AF in minimally adjusted and fully adjusted models (Figure 3 and Table 3). Compared with Quartile 4, there was no association of LA reservoir strain Quartiles 2 and 3 with incident AF. On sensitivity analyses, the association between lower LA reservoir strain and incident AF was consistent with the primary results upon (a) blanking follow-up for the first year after echocardiogram (Supplemental Table 4), (b) complete case analysis (Supplemental Table 5), and (c) evaluation of LA reservoir strain as a continuous variable (Supplemental Table 6). In exploratory analyses, there was modest improvement in discrimination when LA reservoir strain was added to the simple Cohorts for Heart and Aging Research in Genomic Epidemiology–AF (CHARGE-AF) risk score (Supplemental Table 7).

The associations between measures of LV mechanics and incident AF were linear. While lower LV longitudinal strain was associated with incident AF after adjustment for clinical variables and traditional echocardiographic measures, this association was attenuated after further adjustment for LA reservoir strain (Table 4). The association between LV early diastolic strain rate and incident AF was attenuated after adjustment for variables in the CHARGE-AF score (Table 4).

### Association of LA reservoir strain and incident AF in subgroups.

**Figure 4:**
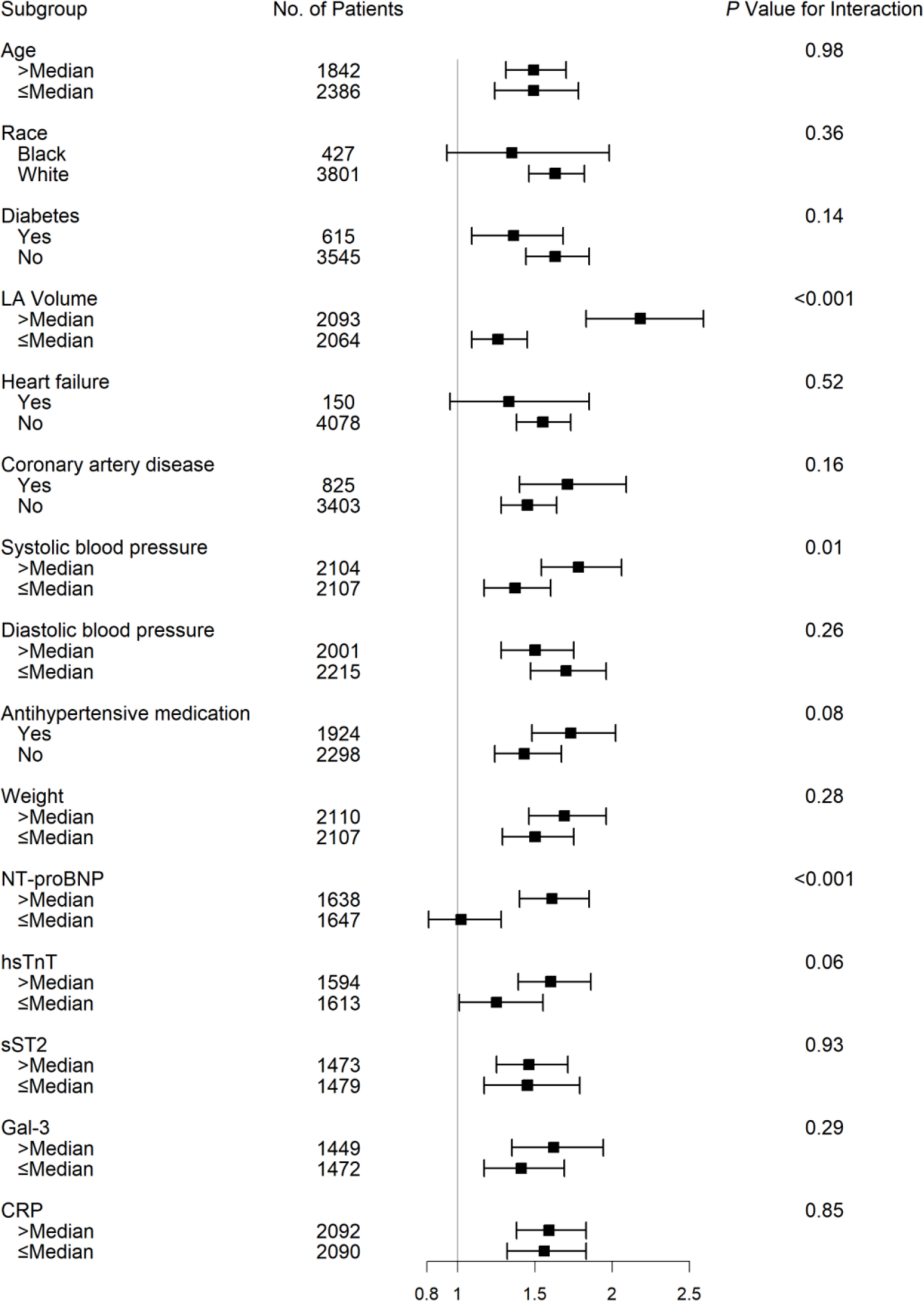
Association of LA reservoir strain and incident AF among subgroups. Cox proportional hazards models incorporating prespecified interaction terms were used to evaluate the associations of LA reservoir strain with incident AF in certain subgroups. CRP, C-reactive protein; Gal-3, galectin 3; hs-TnT, high sensitivity troponin T; LA, left atrial; NT-proBNP, N-terminal pro–B-type natriuretic peptide; sST2, soluble ST2.

The associations of LA reservoir strain with incident AF among prespecified subgroups are shown in Figure 4. The association of LA reservoir strain with incident AF was stronger among (a) individuals with systolic blood pressure above median, (b) participants with baseline LAVs above median compared with below median, and (c) participants with baseline NT-proBNP levels above median compared with below median (Figure 4). The association of LA reservoir strain and incident AF was consistent across all remaining subgroups, including across the spectrum of C-reactive protein (CRP).

## Discussion

In this analysis of an elderly, high-risk cohort with long-term follow-up, we comprehensively describe the longitudinal associations between several baseline indices of cardiac mechanics and incident AF. Participants with lower LA reservoir strain had significantly higher baseline prevalence of several risk factors and cardiovascular comorbidities, and they had baseline echocardiograms with more severe derangements in several indices of LA and LV structure and function. Over the course of 10 years, 11.4% of participants developed AF. Lower LA reservoir strain was associated with incident AF, independent of clinical characteristics, LAV, and LV mechanics. The association between LA reservoir strain and incident AF was particularly strong among subgroups of participants with higher blood pressure, LAVs, and biomarkers of neurohormonal activation. After adjustment for LA reservoir strain, there was no independent association of LV mechanics and incident AF. In aggregate, our findings substantiate the notion that LA myopathy precedes overt AF and suggests that the complex LA myocardial substrate quantified by strain imaging is strongly associated with development of AF.

LA myopathy, defined broadly as any abnormality in LA structure and/or function, has been previously associated with the development of AF. Anatomic remodeling of the LA, leading to increased LA diameter and volume, has been strongly associated with incident AF in several populations (3, 12). Subsequently, associations of various indices of LA function with incident AF have also been investigated. Indeed, echocardiographic parameters of LA function that are dependent upon LA size (i.e., LA emptying fraction and LA function index) are associated with incident AF (13, 14). However, such gross measures of structural or functional remodeling may be a later sign of LA myopathy that may not be as readily reversible once detected and do not offer substantial insight with regard to tissue-level ultrastructural abnormalities. Recently, LA reservoir strain has been associated with incident AF in small cohorts over a relatively short duration of follow-up (8, 15). Additionally, LA strain as measured by cardiac magnetic resonance has also been associated with incident AF (16, 17). Our primary study findings extend the body of evidence supporting the temporal association of subclinical alterations in LA mechanics with incident AF to a large, community-based elderly cohort with high rates of AF over the course of 10 years. Additionally, this study further demonstrates that the unique association of LA reservoir strain with AF is independent of not only clinical variables associated with AF, but also independent of degree of anatomic LA remodeling, LV structure, and LV longitudinal strain. In aggregate, our study raises the possibility that the abnormal LA substrate detected by strain imaging may specifically drive the development of AF.

While the myopathic substrate that drives AF to exist has been previously investigated, LA strain uniquely quantifies LA tissue deformation, providing insights into mechanisms driving AF. LA strain has demonstrated strong correlation with LA fibrosis on cardiac magnetic resonance, which is in line with the known differences in tensile properties of healthy myocardium compared with fibrotic tissue (10). Thus, it is possible that echocardiographic LA strain is reflective, in part, of LA fibrosis. However, LA strain offers further potential insight regarding ultrastructural and functional changes in addition macrostructural derangements (i.e., replacement fibrosis). Indeed, abnormalities in strain are detected before the development of myocardial fibrosis and occurred in concert with cardiomyocyte T-tubule disorganization and impaired calcium cycling (11). Given the known contribution of cardiomyocyte calcium-handling abnormalities in promoting AF (18), it is possible that LA reservoir strain indeed captures the mechanical derangements caused by the underlying process of abnormal calcium cycling. Therefore, LA reservoir strain is a unique, aggregate measure of myocardial function that appears to offer orthogonal information regarding AF risk, independent of traditional measures of LAV. Indeed, the association of LA reservoir strain and AF was significant even after adjustment for LA size among an older cohort with a substantial degree of cardiac remodeling at baseline. Additionally, the correlation between LA reservoir strain and LAV, while significant, was rather modest in our study, and there was wide variation in degree of LA functional impairment for any degree of LA size. It is possible that LA reservoir strain may be a marker of vulnerable myocardial substrate, as opposed to a driver of AF itself. However, the consistent associations of LA reservoir strain with AF in our study, in addition to the known ultrastructural myocardial derangements reflected by LA strain, suggest a mechanistic association between low LA strain and AF. These findings are, thus, important, since they have implications for therapeutic targeting of this LA substrate to potentially reduce AF risk. For example, therapies aimed at improved LA mechanical function (e.g., reduction in LA fibrosis or regulation of LA cardiomyocyte calcium handing) may reduce future AF risk and warrant further investigation.

The association between LA reservoir strain and AF was particularly strong in specific subgroups of participants with higher blood pressure, larger LAVs, and higher biomarkers of neurohormonal activation. As hypertension (19), larger LA size (3), and higher NT-proBNP (20, 21) have been individually associated with incident AF, our findings suggest that LA reservoir strain may be a particularly important tool for risk stratification among those at highest risk for AF. Indeed, LA reservoir strain may serve a useful role in sequential risk stratification among those individuals at high risk for AF based on clinical variables and biomarkers. It is possible that the association between LA reservoir strain and incident AF is stronger in these subgroups because these participants may have subclinical, undetected AF. However, sensitivity analysis that excluded incident AF events occurring within the first year of follow-up yielded consistent results, which makes this less likely.

While the association of LA reservoir strain and incident AF was independent of LV mechanics, we noted that LV mechanics were not independently associated with AF. Specifically, LV longitudinal strain, a sensitive measure of LV systolic function, was not associated with AF upon adjustment for LA reservoir strain. Previous investigations have demonstrated that parameters of LV size and function may be related to development of AF (8, 22). Our findings suggest LA dysfunction specifically is a stronger marker of AF risk and further substantiates the understanding that LA myopathy out of proportion to LV myopathy leads to a high-risk myocardial substrate for AF.

### Strengths and limitations.

Our study has both strengths and limitations. CHS is a large, well-phenotyped, elderly population at substantial risk for cardiac events. AF was ascertained through adjudicated, annual 12-lead electrocardiograms (ECGs) over long-term follow-up; hospital discharge diagnoses; and Medicare claims for AF. While this method may underestimate true incidence of AF, it has been validated in several populations, is easily translatable to clinical practice, and may represent clinically significant AF, as device-detected AF through continuous monitoring is of relatively low yield and uncertain clinical benefit (23). Nonetheless, the possibility of underestimation of AF incidence would tend to bias our results to null. The aim of this study was to evaluate the association of cardiac mechanics with AF; further investigation is required to understand the incremental value added by LA strain to predict AF. The image characteristics at the time of the echocardiogram (30 fps) and the lack of high-fidelity electrocardiographic tracings during the echocardiogram (for accurate detection of the P wave) prevented derivation of strain rates and acquisition of LA conduit strain and LA contractile strain, respectively. Thus, our analysis was focused upon strain measurements that could be readily obtained through the imaging characteristics of CHS echocardiograms (e.g., LA reservoir strain), which has been validated in other cohorts (24). Due to inadequate image quality and lack of echocardiographic data, a number of participants were excluded from the current analysis. While a higher proportion of Black participants were excluded because of inadequate image quality, there was no observed interaction by race or ethnicity on the primary results. Variation in speckle tracking and image quality may have confounded the association of LA reservoir strain and incident AF. However, such variability would be expected to attenuate such associations, and we adjusted for speckle-tracking analyst, field center, and image quality in all analyses.

In an elderly cohort, participants with adverse baseline cardiac mechanics were characterized by a clinical profile of higher comorbidity burden and an echocardiographic profile of more frequent LA and LV structural and functional derangements. Lower LA reservoir strain was independently associated with incident AF over long-term follow-up. The association of LA reservoir strain and incident AF was particularly strong among high-risk subgroups with higher blood pressure, NT-proBNP levels, and LAVs. These findings suggest that mechanical dysfunction of the LA specifically precedes the development of AF and offers insight into the myocardial substrate that drives the development of AF. Therapies targeting this specific LA myocardial substrate may prevent progression to AF.

## Methods

### Study population.

CHS is a prospective cohort of adults (≥65 years) that was initially established to determine risk factors for cardiovascular disease. Information regarding study design, data collection, and definition of comorbid conditions have been previously published (25, 26). Initially, 5201 community-dwelling adults were recruited between 1989 and 1990. A supplemental cohort of 687 participants, predominantly Black individuals, was subsequently enrolled between 1992 and 1993. Participants were recruited across 4 US field centers (Sacramento, California, USA; Hagerstown, Maryland, USA; Winston-Salem, North Carolina, USA; and Pittsburgh, Pennsylvania, USA). From initial recruitment until 1999, participants underwent annual comprehensive examinations that consisted of medical history, laboratory testing, and 12-lead ECGs. These alternated with telephone contact of participants every 6 months. Following completion of in-person examinations, telephone contacts replaced visits and were continued on a semiannual basis. For the present analysis, participants who (a) were free of AF at recruitment and (b) had echocardiograms with speckle-tracking strain analysis of at least 1 cardiac chamber (LA or LV) were included.

### Echocardiographic speckle-tracking analysis.

Comprehensive 2-dimensional (2-D), M-mode, and Doppler echocardiograms were obtained in 1989–1990 and again in 1994–1995, and they served as the baseline scans for the original and supplemental cohorts, respectively. The echocardiograms followed a standardized protocol and were interpreted at core reading centers (27). At the 4 field centers, echocardiograms were recorded onto Super VHS tapes using Toshiba SSH-160A cardiac ultrasound machines. Videotapes were sent to the Echocardiography Reading Center (Irvine, California, USA, for the 1989–1990 echocardiograms, and Washington, DC, for the 1994–1995 echocardiograms), where images were digitized and initial measurements were made using M-mode, 2-D, and Doppler images (28).

From 2016 to 2018, archived CHS echocardiograms were digitized using the TIMS 2000 DICOM system (Foresight Imaging, Chelmsford, Massachusetts, USA), using methods developed by our group for a similar analysis done in the Hypertension Genetic Epidemiology Study (HyperGEN Study) (24, 29). Cine loops of 2–4 cardiac cycles from the apical 4-chamber view were digitized at a frame rate of 30 frames per second and stored offline in DICOM format (Northwestern University). Speckle-tracking echocardiography was subsequently performed for strain analysis using specific software (TOMTEC Cardiac Performance Analysis, v4.5) by 5 experienced readers. All echocardiograms were assigned chamber-specific image quality scores (from 0 to 4) based on degree of visualization of the myocardium and cardiac structures, as described previously (24). Speckle-tracking measurements were made with the R-R wave ECG gating to define the cardiac cycle. The LA endocardial border was traced manually in the apical 4-chamber view for creation of the LA longitudinal strain curve. Six segments of the LA were identified by strain software; segments that did not track appropriately were removed from the analysis, and the average of the remaining segments was generated. Given that the ventricular cycle was the reference point, all LA strain values were reported as positive absolute percentages. LA reservoir strain was defined as the peak average LA strain and corresponded to ventricular systole. LV longitudinal strain and LV early diastolic strain rate were derived through similar methods after manually tracing the LV endocardial border in the apical 4-chamber view. Reproducibility of strain measures is reported in Supplemental Table 8. LA endocardial borders were traced during LV end-systole and LAV was calculated using the biplane method. LV mass was derived using the Devereux formula (30). The primary measurements of interest in this study were LA reservoir strain, LV longitudinal strain, and LV early diastolic strain rate.

### Biomarker profiling.

Serum levels of NT-proBNP, hs-TnT, sST2, Gal-3, and CRP were obtained at baseline examinations. The manufacturers for the assays for each biomarker include the following: NT-proBNP (Elecsys2010 analyzer, Roche Diagnostics); hs-TnT (Elecsys 2010 analyzer, Roche Diagnostics); sST2 (Presage ST2 ELISA, Critical Diagnostics); Gal-3 (BG Medicine ELISA); and CRP (University of Vermont ELISA, Burlington, Vermont, USA) (31).

### Ascertainment of AF.

Incident AF in CHS was defined as presence of AF on 12-lead ECG obtained during annual study visit, hospital discharge diagnoses of AF (ICD-9 codes 427.3, 427.31, or 427.32), or Medicare claims for AF from inpatient, outpatient, and physician claims. ECGs were read and AF was adjudicated by a centralized ECG reading center (32). Upon review of medical records (including ECGs), ICD-9 discharge codes demonstrate a positive predictive value of 98.6% for diagnosing AF (33). Additionally, previous investigation of CHS has examined the results of 24-hour Holter monitoring among 819 participants, which demonstrated that only 0.1% (*n* = 1) of participants had AF that was not captured using the above criteria (34). We set the follow-up period to 10 years from study baseline for each cohort to assess incident AF events.

### Statistics.

Baseline covariates for each participant were defined at the time of baseline echocardiography (i.e., 1989–1990 for the original cohort, and 1994–1995 for the supplemental cohort). Baseline characteristics by quartile of LA reservoir strain were compared using χ^2^ tests for categorical variables and 1-way ANOVA or Kruskal-Wallis tests (depending on distribution) for continuous variables. The association of LAV with LA reservoir strain was assessed using a linear regression model. LAVs were log-transformed to maintain homoscedasticity of residuals in the regression model.

Multivariable Cox proportional hazards regression models evaluated the associations of each echocardiographic measure of cardiac mechanics with incident AF. The proportionality of hazards assumption was confirmed for all models by Schoenfeld goodness-of-fit procedures. We assessed the potential nonlinear associations of indices of cardiac mechanics and hazard of incident AF using restricted cubic splines in Cox proportional hazards regression. In the absence of nonlinearity, we assessed the association of indices of cardiac mechanics as continuous variables (per 1 SD lower) with incident AF. Based on strength of nonlinearity, we assessed the association of LA reservoir strain with incident AF using quartiles of LA reservoir strain.

For all Cox regression models, Model 1 adjusted for field center, speckle-tracking analyst, and image quality. Model 2 further adjusted for clinical variables within the simple CHARGE-AF risk score and sex. The simple CHARGE-AF risk score is composed of 11 clinical variables (age, race, height, weight, systolic blood pressure, diastolic blood pressure, antihypertensive medication use, current smoker, DM, HF, and myocardial infarction) and has exhibited excellent test characteristics for AF prediction (19). Model 3 further adjusted for the following echocardiographic variables: LAV, LV mass, LVEF, and septal early diastolic tissue velocity (e’ velocity). Model 4 further adjusted for either LA reservoir strain if LV strain measures represented the exposure variables or LV longitudinal strain if the exposure variable was LA reservoir strain.

Kaplan-Meier curves were used to display AF status by quartile of LA reservoir strain over time. Interaction analyses were performed to evaluate the association of LA reservoir strain and incident AF among certain subgroups using interaction terms for: age, race, diabetes, heart failure, prior myocardial infarction, systolic blood pressure, diastolic blood pressure, weight, antihypertensive medication use, NT-proBNP, hs-TnT, sST2, Gal-3, and CRP. We performed 3 sensitivity analyses to evaluate the consistency of our findings. We evaluated the association of LA reservoir strain and incident AF with a blanking period of 1 year from time of echocardiography using Cox proportional hazards regression to minimize the potential for reverse causation (i.e., undiagnosed AF resulting in worse LA or LV strain). We also evaluated the association of LA reservoir strain with incident AF among a complete case cohort with all clinical and echocardiographic covariates available. Finally, we assessed the association of LA reservoir as a continuous variable (per 1 SD lower) with incident AF. In exploratory analyses, we assessed the incremental contribution of LA reservoir strain to the CHARGE-AF risk score in predicting AF, which is composed of readily available clinical variables (19). We examined the change in model discrimination after addition of such measures of cardiac mechanics to the CHARGE-AF risk score. We compared model discrimination using Harrell’s C-statistic. We compared calibration of models using the Greenwood-Nam-D’Agostino (GND) statistic. Analyses were carried out in SAS and R version 3.5.0 (R Foundation for Statistical Computing).

### Study approval.

The study protocol was approved by the IRBs of the coordinating center (University of Washington, Seattle, Washington, USA) and each of the CHS field centers. All participants provided written informed consent.

## Author contributions

RBP contributed study concept/design, analysis and interpretation of data, and drafting the manuscript. JAD contributed study design, analysis and interpretation of data, and critical manuscript revisions for important intellectual content. MH contributed analysis and interpretation of data and critical manuscript revisions for important intellectual content. HP contributed acquisition of data and critical manuscript revisions for important intellectual content. JC contributed acquisition of data and critical manuscript revisions for important intellectual content. JG contributed acquisition and interpretation of data, as well as critical manuscript revisions for important intellectual content. JRK contributed study design, interpretation of data, and critical manuscript revisions for important intellectual content. GMM contributed interpretation of data and critical manuscript revisions for important intellectual content. MPT contributed interpretation of data and critical manuscript revisions for important intellectual content. RD contributed interpretation of data and critical manuscript revisions for important intellectual content. SRH contributed interpretation of data and critical manuscript revisions for important intellectual content. BMP contributed study design, acquisition and interpretation of data, and critical manuscript revisions for important intellectual content. SJS contributed study concept/design; acquisition, analysis, and interpretation of data; and drafting the manuscript.

## Supplementary Material

Supplemental data

ICMJE disclosure forms

## Figures and Tables

**Table 4:**
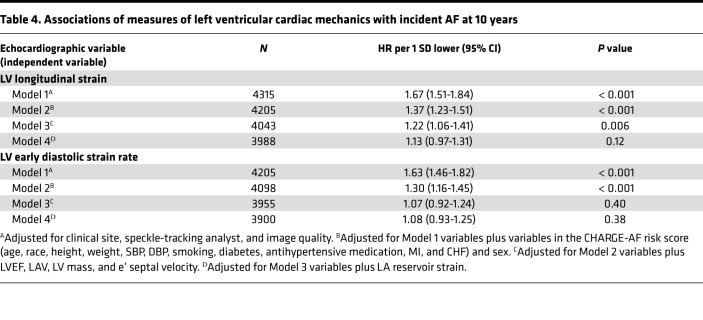
Associations of measures of left ventricular cardiac mechanics with incident AF at 10 years

**Table 3:**
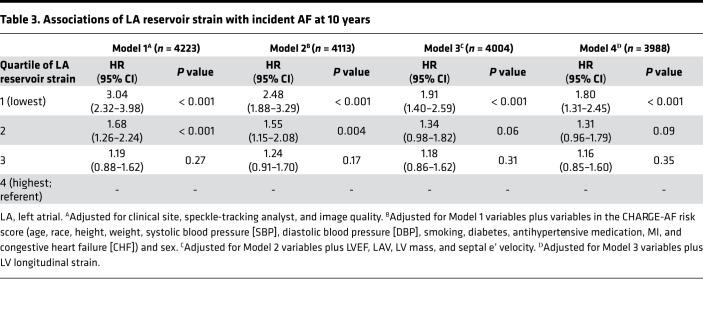
Associations of LA reservoir strain with incident AF at 10 years

**Table 1:**
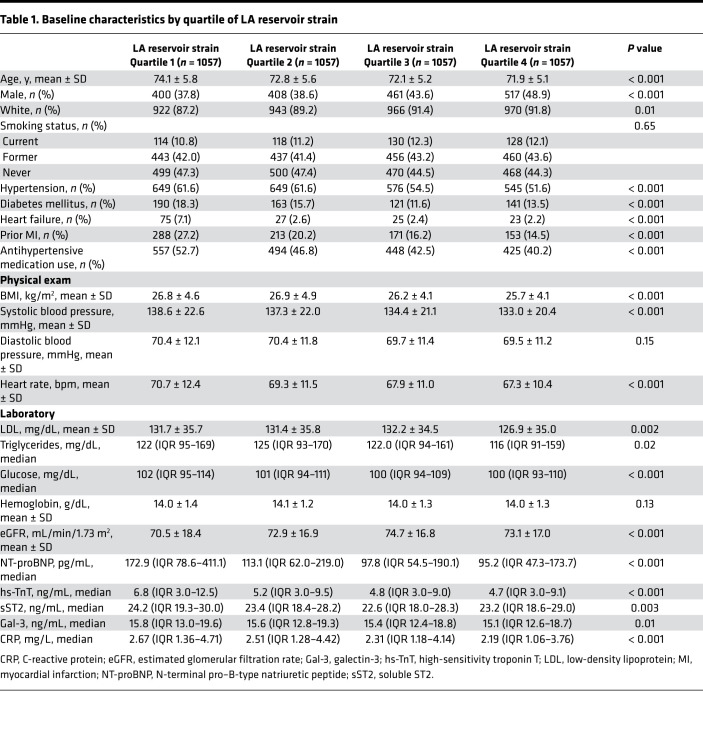
Baseline characteristics by quartile of LA reservoir strain

**Table 2:**
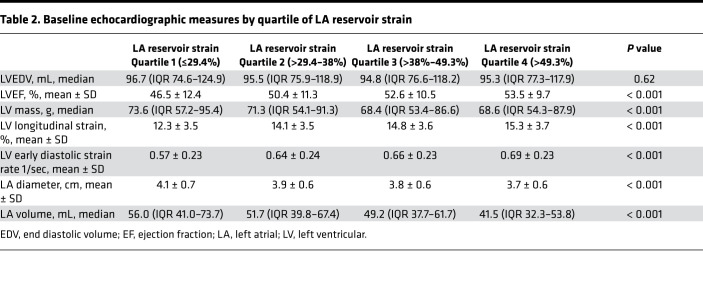
Baseline echocardiographic measures by quartile of LA reservoir strain

## Version 1 09/10/2020

In-Press Preview

## Version 2 10/02/2020

Electronic publication

## Version 3 10/02/2020

The abbreviation for HR (Hazard Ratio) was corrected in the abstract.
