# Supplementary material for: Characterization of cardiac mechanics and incident atrial fibrillation in participants of the Cardiovascular Health Study
Source: JCI Insight. 2020 Oct 2;5(19):e141656. doi: 10.1172/jci.insight.141656 (PMC7566702; doi:10.1172/jci.insight.141656)
Supplement: Supplemental data [file jciinsight-5-141656-s247.pdf]

Online Appendix

Supplemental Table 1. Characteristics of Participants Without Atrial Fibrillation at Baseline Excluded from Analysis.

Characteristic	Included Participants (n=4341)	Excluded Participants (n=1371)
Age, y	72.8 (5.5)	73.6 (5.8)
Male, n (%)	1848 (42.6)	549 (40.0)
White, n (%)	3899 (89.8)	880 (64.2)
Smoking Status, n (%)		
Current	506 (11.7)	154 (12.3)
Former	1839 (42.4)	1016 (41.4)
Never	1990 (45.9)	517 (41.2)
Hypertension, n (%)	2481 (57.2)	584 (46.5)
Diabetes mellitus, n (%)	632 (14.8)	243 (20.2)
Heart failure, n (%)	158 (3.6)	98 (7.2)
Prior MI, n (%)	843 (19.4)	282 (20.8)
Anti-hypertensive medication use, n (%)	1971 (45.5)	642 (51.2)
**Physical Exam**		
BMI, kg/m^2, mean (SD)	26.3 (4.5)	27.8 (5.3)
Systolic blood pressure, mmHg (SD)	135.8 (21.6)	137.2 (21.4)
Diastolic blood pressure, mmHg (SD)	70.0 (11.6)	72.1 (11.3)
**Laboratory**		
LDL, mg/dL, mean (SD)	130.5 (35.3)	129.6 (36.4)
Hemoglobin, g/dL, mean (SD)	14.0 (1.3)	13.9 (1.7)
eGFR, mL/min/1.73m^2, mean (SD)	72.8 (17.4)	74.8 (15.7)

BMI = body mass index; eGFR = estimated glomerular filtration rate; Gal-3 = galectin-3; hsTnT = high-sensitivity troponin T; LDL = low-density lipoprotein; MI = myocardial infarction; NT-proBNP = N-terminal pro-B-type natriuretic peptide; sST2 = soluble ST2

Supplemental Table 2. Baseline Characteristics by Quartile of LV Longitudinal Strain.

	**LV Longitudinal strain Quartile 1 (n=1080)**	**LV Longitudinal strain Quartile 2 (n=1080)**	**LV Longitudinal strain Quartile 3 (n=1080)**	**LV Longitudinal strain Quartile 4 (n=1080)**	**P value**
Age, y, mean ±SD	73.5±5.8	72.9±5.5	72.7±5.6	71.9±5.0	<0.001
Male, n (%)	602 (55.7)	499 (46.2)	421 (39.0)	312 (28.9)	<0.001
White, n (%)	939 (86.9)	960 (88.9)	993 (91.9)	987 (91.4)	0.001
Smoking Status, n (%)					0.27
Current	123 (11.4)	134 (12.4)	116 (10.8)	132 (12.2)	
Former	486 (45.1)	459 (42.5)	449 (41.6)	435 (40.3)	
Never	468 (43.5)	486 (45.0)	514 (47.6)	512 (47.5)	
Hypertension, n (%)	691 (64.0)	642 (59.5)	597 (55.4)	541 (50.1)	<0.001
Diabetes mellitus, n (%)	236 (22.3)	160 (15.1)	128 (12.0)	105 (9.8)	<0.001
Heart failure, n (%)	83 (7.7)	23 (2.1)	27 (2.5)	19 (1.8)	<0.001
Prior MI, n (%)	332 (30.7)	200 (18.5)	160 (14.8)	141 (13.1)	<0.001
Anti-hypertensive medication use, n (%)	579 (53.7)	486 (45.1)	474 (43.9)	421 (39.1)	<0.001
**Physical Exam**					
BMI, kg/m^2, mean ±SD	27.2±4.7	26.6±4.6	26.1±4.2	25.4±4.1	<0.001
Systolic blood pressure, mmHg, mean ±SD	139.0±22.0	136.6±21.2	135.0±21.4	132.7±21.2	<0.001
Diastolic blood pressure, mmHg, mean ± SD	71.5±12.0	70.5±11.8	69.8±11.1	68.4±11.3	<0.001
Heart rate, bpm, mean ±SD	71.6±12.4	69.1±11.1	68.4±11.0	66.0±10.4	<0.001
**Laboratory**					
LDL, mg/dL, mean ±SD	130.0±35.3	130.0±35.4	129.8±35.1	132.3±35.3	0.29
Triglycerides, mg/dL, median (IQR)	131.0 (99.0-177.8)	124.0 (95.0-169.0)	118.0 (91.0-160.0)	112.0 (88.0-151.0)	<0.001
Glucose, mg/dL, median (IQR)	104.0 (96.0-119.0)	101.0 (94.0-111.0)	100.0 (94.0-109.0)	98.0 (92.0-107.0)	<0.001
Hemoglobin, g/dL, mean ± SD	14.2±1.4	14.1±1.3	14.0±1.3	13.8±1.2	<0.001
eGFR, mL/min/1.73m^2, mean ±SD	70.5±18.5	72.1±17.7	73.3±17.0	74.9±16.0	<0.001
NT-proBNP, pg/mL, median (IQR)	141.2 (64.4-359.5)	116.8 (57.3-221.9)	99.5 (54.3-187.2)	109.4 (61.5-199.0)	<0.001
hsTnT, ng/mL, median (IQR)	7.3 (3.0-12.9)	5.8 (3.0-10.0)	4.6 (3.0-9.0)	3.9 (3.0-8.0)	<0.001
sST2, ng/mL, median (IQR)	25.2 (20.1-31.5)	23.5 (18.5-28.6)	22.9 (18.6-28.8)	22.0 (17.7-26.7)	<0.001

Gal-3, ng/mL, median (IQR)	15.5 (12.6-19.5)	15.8 (12.9-19.7)	15.4 (12.8-18.6)	15.3 (12.5-19.1)	0.31

BMI = body mass index; eGFR = estimated glomerular filtration rate; Gal-3 = galectin-3; hsTnT = high-sensitivity troponin T; LDL = low-density lipoprotein; LV = left ventricular MI = myocardial infarction; NT-proBNP = N-terminal pro-B-type natriuretic peptide; sST2 = soluble ST2

Supplemental Table 3. Baseline Characteristics by Quartile of LV Early Diastolic Strain Rate.

	LV Early Diastolic Strain Rate Quartile 1 (n=1058)	LV Early Diastolic Strain Rate Quartile 2 (n=1055)	LV Early Diastolic Strain Rate Quartile 3 (n=1048)	LV Early Diastolic Strain Rate Quartile 4 (n=1049)	P value
Age, y, mean ±SD	74.0±5.9	72.9±5.5	72.3±5.4	71.6±4.9	<0.001
Male, n (%)	580 (54.8)	473 (44.8(	413 (39.4)	313 (29.8)	<0.001
White, n (%)	937 (88.6)	940 (89.1)	940 (89.7)	968 (92.3)	0.13
Smoking Status, n (%)					0.22
Current	109 (10.3)	118 (11.2)	127 (12.1)	140 (13.4)	
Former	479 (45.3)	445 (42.3)	430 (41.1)	434 (41.4)	
Never	469 (44.4)	489 (46.5)	490 (46.8)	474 (45.2)	
Hypertension, n (%)	677 (64.0)	640 (60.8)	579 (55.3)	510 (48.7)	<0.001
Diabetes mellitus, n (%)	205 (19.7)	157 (15.2)	134 (13.0)	104 (10.0)	<0.001
Heart failure, n (%)	68 (6.4)	28 (2.7)	21 (2.0)	24 (2.3)	<0.001
Prior MI, n (%)	303 (28.6)	194 (18.4)	159 (15.2)	143 (13.6)	<0.001
Anti-hypertensive medication use, n (%)	551 (52.2)	499 (47.3)	450 (43.0)	398 (37.9)	<0.001
**Physical Exam**					
BMI, kg/m^2, mean ±SD	27.2±4.4	26.5±4.5	26.1±4.4	25.4±4.3	<0.001
Systolic blood pressure, mmHg, mean ±SD	138.9±21.8	137.4±21.5	135.0±21.0	131.5±20.8	<0.001
Diastolic blood pressure, mmHg, mean ± SD	71.0±11.9	70.7±11.6	69.9±11.4	68.4±11.1	<0.001
Heart rate, bpm, mean ±SD	68.2±10.6	68.3±10.9	69.5±11.9	69.2±12.2	0.02
**Laboratory**					
LDL, mg/dL, mean ±SD	129.2±35.3	131.5±35.1	130.6±35.6	131.4±35.4	0.42
Triglycerides, mg/dL, median (IQR)	129.0 (97.0-177.0)	123.0 (95.0-169.0)	119.0 (91.0-161.0)	112.0 (88.0-152.0)	<0.001
Glucose, mg/dL, median (IQR)	103.0 (95.0-115.0)	101.0 (94.0-112.0)	100.0 (94.0-109.0)	98.0 (92.0-107.0)	<0.001
Hemoglobin, g/dL, mean ± SD	14.2±1.4	14.1±1.3	14.0±1.3	13.8±1.3	<0.001
eGFR, mL/min/1.73m^2, mean ±SD	69.7±18.4	72.2±17.0	74.5±16.8	74.8±16.5	<0.001
NT-proBNP, pg/mL, median (IQR)	141.8 (69.6-309.3)	117.3 (58.9-214.5)	96.3 (49.1-186.7)	105.3 (59.5-199.6)	<0.001
hsTnT, ng/mL, median (IQR)	7.0 (3.2-11.9)	5.5 (3.0-10.2)	4.9 (3.0-9.2)	3.9 (3.0-8.2)	<0.001

sST2, ng/mL, median (IQR)	24.7 (19.8-30.9)	23.7 (18.9-29.1)	22.9 (18.4-28.2)	21.9 (17.6-27.8)	<0.001
Gal-3, ng/mL, median (IQR)	15.7 (12.6-20.4)	15.4 (12.5-18.6)	15.5 (12.7-18.9)	15.4 (12.7-19.0)	0.17

BMI = body mass index; eGFR = estimated glomerular filtration rate; Gal-3 = galectin-3; hsTnT = high-sensitivity troponin T; LDL = low-density lipoprotein; MI = myocardial infarction; NT-proBNP = N-terminal pro-B-type natriuretic peptide; sST2 = soluble ST2;

Supplemental Table 4. Association of LA Reservoir Strain and AF With Blanking for 1 Year After Echocardiogram.

	Model 1* (N=4164)		Model 2† (N=4055)		Model 3†† (N=3948)		Model 4§ (N=3932)	
**Quartile of LA reservoir strain**	**HR (95% CI)**	**P value**	**HR (95% CI)**	**P value**	**HR (95% CI)**	**P value**	**HR (95% CI)**	**P value**
1 (lowest)	3.00 (2.28-3.93)	<0.001	2.46 (1.86-3.26)	<0.001	1.92 (1.41-2.61)	<0.001	1.79 (1.31-2.46)	<0.001
2	1.65 (1.23-2.21)	<0.001	1.53 (1.13-2.06)	0.006	1.33 (0.98-1.82)	0.07	1.30 (0.95-1.78)	0.10
3	1.20 (0.88-1.63)	0.24	1.25 (0.92-1.71)	0.16	1.19 (0.87-1.65)	0.28	1.18 (0.85-1.62)	0.32
4 (highest; referent)	-	-	-	-	-	-	-	-

LA = left atrial; LV = left ventricular

* Adjusted for clinical site, speckle-tracking analyst, and image quality
† Adjusted for Model 1 variables plus variables in the CHARGE-AF Risk score (age, race, height, weight, SBP, DBP, smoking, diabetes, anti-hypertensive medication, MI, and CHF) and sex
†† Adjusted for Model 2 variables plus LVEF, LAV, LV mass, and e' septal velocity
§ Adjusted for Model 3 variables plus LV longitudinal strain

Supplemental Table 5. Association of LA Reservoir Strain with Incident AF: Complete Case Analysis.

	**Model 1*** (N=3890)		**Model 2†** (N=3890)		**Model 3††** (N=3890)		**Model 4§** (N=3890)	
**Quartile of LA reservoir strain**	**HR (95% CI)**	**P value**	**HR (95% CI)**	**P value**	**HR (95% CI)**	**P value**	**HR (95% CI)**	**P value**
1 (lowest)	3.02 (2.28-4.00)	<0.001	2.39 (1.79-3.20)	<0.001	1.77 (1.30-2.41)	<0.001	1.70 (1.24-2.32)	<0.001
2	1.64 (1.21-2.22)	0.001	1.47 (1.08-2.01)	0.01	1.21 (0.88-1.66)	0.24	1.19 (0.86-1.63)	0.30
3	1.22 (0.89-1.68)	0.23	1.25 (0.91-1.72)	0.17	1.16 (0.84-1.59)	0.38	1.14 (0.83-1.58)	0.43
4 (highest; referent)	-	-	-	-	-	-	-	-

* Adjusted for clinical site, speckle-tracking analyst, and image quality
† Adjusted for Model 1 variables plus variables in the CHARGE-AF Risk score (age, race, height, weight, SBP, DBP, smoking, diabetes, anti-hypertensive medication, MI, and CHF) and sex
†† Adjusted for Model 2 variables plus LVEF, LAV, LV mass, and e' septal velocity
§ Adjusted for Model 3 variables plus LV longitudinal strain

Supplemental Table 6. Association of LA reservoir strain (continuous variable) with incident AF.

Echocardiographic Variable (independent variable)	N	HR per 1-SD lower (95% CI)	P value
**LA reservoir strain**			
Model 1*	4223	1.63 (1.46-1.82)	<0.001
Model 2[†]	4113	1.46 (1.31-1.63)	<0.001
Model 3[††]	4004	1.30 (1.15-1.46)	<0.001
Model 4§	3988	1.26 (1.12-1.43)	<0.001

* Adjusted for clinical site, speckle-tracking analyst, and image quality
[†] Adjusted for Model 1 variables plus variables in the CHARGE-AF Risk score (age, race, height, weight, SBP, DBP, smoking, diabetes, anti-hypertensive medication, MI, and CHF) and sex
[††] Adjusted for Model 2 variables plus LVEF, LAV, LV mass, and e’ septal velocity
§ Adjusted for Model 3 variables plus LV longitudinal strain

Supplemental Table 7. Model characteristics for 10-year AF prediction.

	**CHARGE-AF Score (Simple)**	**CHARGE-AF (Simple) + LA reservoir strain**	**P value**
C-statistic (95% CI)	0.781 (0.754-0.808)	0.788 (0.761-0.815)	3.3×10^{-10}
GND chi-square (P value)	3.39 (0.85)	4.10 (0.77)	

Supplemental Table 8. Intra- and Interobserver Variability of Indices of Cardiac Mechanics

Parameter	Mean ± SD	ICC (95% CI)	Mean bias (95% CI)	CV
**Intraobserver variability (n=46)**				
LV longitudinal systolic strain, %	13.9 ± 4.06	0.98 (0.96, 0.99)	0.05 (-0.31, 0.41)	7.0%
LV early diastolic strain rate, s^{-1}	0.64 ± 0.26	0.95 (0.92, 0.97)	-0.02 (-0.005, 0.02)	16.1%
LA reservoir strain, %	42.9 ± 14.9	0.99 (0.98, 0.99)	0.05 (-0.72, 0.82)	4.6%
**Interobserver variability (n=96)**				
LV longitudinal systolic strain, %	14.2 ± 4.0	0.94 (0.91, 0.96)	-0.58 (-0.97, -0.20)	9.8%
LV early diastolic strain rate, s^{-1}	0.64 ± 0.22	0.90 (0.84, 0.93)	-0.04 (-0.07, -0.02)	16.1%
LA reservoir strain, %	40.4 ± 16.9	0.96 (0.94, 0.97)	0.56 (-0.71, 1.84)	11.5%

LV=left ventricular; LA=left atrial
